# Effects of Expression of *Streptococcus pneumoniae* PspC on the Ability of *Streptococcus mitis* to Evade Complement-Mediated Immunity

**DOI:** 10.3389/fmicb.2021.773877

**Published:** 2021-11-22

**Authors:** Helina Marshall, Ricardo J. José, Mogens Kilian, Fernanda C. Petersen, Jeremy S. Brown

**Affiliations:** ^1^Centre for Inflammation and Tissue Repair, UCL Respiratory, Department of Medicine, Royal Free and University College Medical School, University College London, London, United Kingdom; ^2^Wellcome-Wolfson Institute for Experimental Medicine, Queen’s University Belfast, Belfast, United Kingdom; ^3^Department of Biomedicine, Faculty of Health, Aarhus University, Aarhus, Denmark; ^4^Department of Oral Biology, Faculty of Dentistry, University of Oslo, Oslo, Norway

**Keywords:** *Streptococcus pneumoniae*, *Streptococcus mitis*, PspC, complement, Factor H

## Abstract

*Streptococcus pneumoniae* and *Streptococcus mitis* are genetically closely related and both frequently colonise the naso-oropharynx, yet *S. pneumoniae* is a common cause of invasive infections whereas *S. mitis* is only weakly pathogenic. We hypothesise that sensitivity to innate immunity may underlie these differences in virulence phenotype. We compared the sensitivity of *S. pneumoniae* and *S. mitis* strains to complement-mediated immunity, demonstrating *S. mitis* strains were susceptible to complement-mediated opsonophagocytosis. *S. pneumoniae* resistance to complement is partially dependent on binding of the complement regulator Factor H by the surface protein PspC. However, *S. mitis* was unable to bind factor H. The *S. pneumoniae* TIGR4 strain *pspC* was expressed in the *S. mitis* SK142 strain to create a *S. mitis pspC^+^* strain. Immunoblots demonstrated the *S. mitis pspC^+^* strain expressed PspC, and flow cytometry confirmed this resulted in Factor H binding to *S. mitis*, reduced susceptibility to complement and improved survival in whole human blood compared to the wild-type *S. mitis* strain. However, in mouse models the *S. mitis pspC^+^* strain remained unable to establish persistent infection. Unlike *S. pneumoniae* strains, culture in serum or blood did not support increased CFU of the *S. mitis* strains. These results suggest *S. mitis* is highly sensitive to opsonisation with complement partially due to an inability to bind Factor H, but even when complement sensitivity was reduced by expression of *pspC*, poor growth in physiological fluid limited the virulence of *S. mitis* in mice.

## Introduction

The nasopharyngeal commensal *Streptococcus pneumoniae* is also a frequent cause of pneumonia, septicaemia, and meningitis, and is one of the most common causes of death due to a microorganism globally (Fitzwater et al., 2012). *Streptococcus mitis* is the closest genetic relative of *S. pneumoniae* and is also a respiratory tract commensal, although more often found in the oropharynx rather than the nasopharynx (Frandsen et al., 1991; Wan et al., 2001). Unlike *S. pneumoniae*, *S. mitis* has a low virulence potential and rarely causes invasive disease (Marron et al., 2000; Han et al., 2006). Comparative genomics has identified a large number of genes specific for *S. pneumoniae* strains many of which are likely to contribute to the differences in virulence potential to *S. mitis* (Kilian and Tettelin, 2019), but why these closely related species differ so markedly in virulence potential remains unclear. The most important *S. pneumoniae* virulence factor is its carbohydrate capsule, of which there are multiple different structures divided into 100 + serotypes that vary in their virulence potential (Brueggemann et al., 2004). Recent work has shown that many *S. mitis* strains also express a capsule, some of which are structurally identical to *S. pneumoniae* capsular serotypes (Skov Sørensen et al., 2016). We have previously expressed the *S. pneumoniae* TIGR4 strain serotype (ST) 4 capsule, a virulent capsular serotype, in an *S. mitis* strain and shown that this improves immune evasion (Rukke et al., 2014). However, unlike the *S. pneumoniae* TIGR4 strain, the *S. mitis* ST4 strain was still unable to cause sustained infection in mice. These data indicate that the differences in the virulence potential of *S. pneumoniae* and *S. mitis* are not related to just expression of a capsule but must reflect additional differences between the species.

A major component of innate immunity against *S. pneumoniae* is the complement system, a series of roughly 30 circulating serum and cell surface proteins arranged into three enzyme cascades termed the classical, alternative, and mannan binding lectin (MBL) pathways. These lead to the formation of the C3 convertases, which cleave C3 to generate the opsonin and primary effector molecule of the pathway, C3b (Walport, 2001). C3b covalently binds to the surface of *S. pneumoniae* and potently increases their susceptibility to phagocytosis by neutrophils and macrophages (Yuste et al., 2008). In mice, both the classical and alternative complement pathways are important for immunity against *S. pneumoniae* infection (Brown et al., 2002), and this is confirmed by the massively increased incidence of *S. pneumoniae* infection in those with complement pathway deficiencies (Yuste et al., 2007, 2008). Evasion of complement mediated immunity is a key virulence determinant for *S. pneumoniae*, and is mediated by both the capsule and multiple proteins including the choline binding cell wall protein PspC (Dave et al., 2001; Ren et al., 2003; Yuste et al., 2005; Li et al., 2007; Ogunniyi et al., 2009). We and others have shown that loss of the capsule markedly increases opsonisation of *S. pneumoniae* with C3b, and that the sensitivity of different *S. pneumoniae* strains is partly related to capsular serotype (Weinberger et al., 2009; Hyams et al., 2010b, 2013). Binding Factor H (FH), a negative regulator of alternative complement pathway activity, is a method of immune evasion used by several bacterial pathogens to reduce bacterial opsonisation with C3b, including *S. pneumoniae*. FH binding to *S. pneumoniae* is mainly mediated by PspC, which binds FH in a conformation that exposes a second binding site for C3b thereby doubling the affinity of factor H for C3b and causing a fivefold increase in its ability to degrade C3 convertase (Herbert et al., 2015). As a consequence, loss of PspC also increases *S. pneumoniae* sensitivity to complement (Yuste et al., 2010; Hyams et al., 2013). There are many allelic variants of PspC, and FH binding affinity differs between these variants (Janulczyk et al., 2000; Lu et al., 2006). Importantly, resistance to complement and neutrophil phagocytosis between *S. pneumoniae* strains both correlate strongly with the degree of FH binding to PspC, which in turn correlates with the invasive potential of different strains and serotypes (Hyams et al., 2013). PspC is also known to mediate adherence of *S. pneumoniae* to epithelial cells, and to promote *S. pneumoniae* translocation across epithelial layers and the blood brain barrier (Rosenow et al., 1997; Zhang et al., 2000; Iovino et al., 2017). Overall, PspC is a key determinant of the invasive potential of *S. pneumoniae*. In almost all the *S. mitis* strains sequenced to date, no homologue of *pspC* has been identified, suggesting that the lack of PspC could be another reason why *S. mitis* has low virulence potential. Furthermore, little is known of the susceptibility of *S. mitis* to complement in general.

Here we have compared resistance to complement and neutrophil phagocytosis of encapsulated *S. pneumoniae* and *S. mitis* strains, including three strains of each species that express identical capsular serotypes. We have then expressed *pspC* from the *S. pneumoniae* TIGR4 strain in the *S. mitis* strain SK142 to test the hypothesis that the lack of PspC is why *S. mitis* is sensitive to complement and has a low virulence potential.

## Materials and Methods

### Bacterial Strains and Culture Conditions

The strains of *S. pneumoniae* and *S. mitis* used in this study are listed in Table 1, some of which have been previously described (Camberlein et al., 2015; Kilian and Tettelin, 2019). Bacteria were cultured at 37°C in 5% CO_2_ in air on Colombia blood agar (Oxoid, United Kingdom) plates supplemented with 5% defibrinated horse blood (TCS Biosciences, United Kingdom) or in Todd-Hewitt broth supplemented with 0.5% yeast extract (THY) (Oxoid, United Kingdom). Growth in liquid medium was assessed by optical density. Bacterial stocks were grown to approximately mid-log phase and stored in 10% glycerol as single-use aliquots.

**TABLE 1:** Bacterial strains used for this study.

**Species**	**Strain**	**Serotype**
*S. pneumoniae*	TIGR4	4
	SK618	45
	1095/39	36
	SK1442	19C
	TIGR4Δ	Unencapsulated
*S. mitis*	NCTC12261 (SK142)	Unknown
	SK564	19C
	SK1126	36
	CCUG62644	45
	SK142 *pspC*^+^	Unknown

### Growth Assessments

Under sterile conditions, 1 × 10^6^ CFU/ml were inoculated into 1 ml of THY broth in a sterile cuvette and incubated at 37°C for 8 h. The OD was measured every hour at 600 nm, following agitation using a pipette. Each strain was tested in triplicate. For assessing growth in serum, 2 × 10^6^ CFU of each *S. pneumoniae* or *S. mitis* strain were inoculated into 1 ml of serum that had previously been heat inactivated by incubation at 56°C for 30 min or in fresh human blood. The blood was prevented from clotting using a final concentration of 0.1 M sodium citrate. After a 4-h incubation at 37°C, 5% CO_2_, serial dilutions were plated onto Colombia blood agar, incubated overnight and colonies counted to calculate bacterial numbers measured as CFU/ml. Data are presented as the percentage of CFU per ml compared to the initial inoculum count.

### Complement C3 Deposition and Factor H Binding Assay

Complement C3 and FH interactions with *S. pneumoniae* and *S. mitis* were assessed using established flow cytometry assays as previously described (Brown et al., 2002; Hyams et al., 2010a, 2011, 2013). Briefly, 5 × 10^6^ CFU of *S. pneumoniae* and *S. mitis* were pelleted and resuspended in either baby rabbit complement (BRC) or pooled human sera and incubated at 37°C for 20 min. Samples were then washed twice with 0.1% PBS Tween-20. For FH binding, a sheep anti-factor H primary antibody was added at a 1:300 dilution and incubated for 30 min before washing in PBS 0.1% Tween-20 and addition of FITC-labelled donkey anti-sheep secondary antibody (FH binding) or a fluorescein-conjugated goat IgG fraction to rabbit complement C3 (C3b/iC3b deposition). After incubation on ice for 30 min and further washes with PBS 0.1% Tween-20 samples were fixed with 10% neutral buffered formalin and analysed by flow cytometry using a BD FACSVerse flow cytometer.

### Neutrophil Opsonophagocytosis

Phagocytosis was investigated using an established flow cytometry assay (Lu et al., 2008; Hyams et al., 2010a). Briefly, *S. pneumoniae* and *S. mitis* fluorescently labelled with 6-carboxyfluorescein succinimidyl ester (FAMSE; Molecular Probes) were incubated in pooled human serum heat-inactivated (56°C for 30 min), pooled human serum or baby rabbit complement for 30 min at 37°C then added to 10^5^ neutrophils extracted from fresh human blood at a multiplicity of infection (MOI) of 10–1, incubated for 30 min, then analysed using a BD FACSVerse flow cytometer. A minimum of 10,000 cells were analysed by flow cytometry, and the results are presented as a fluorescence index (FI: the percentage of positive bacteria multiplied by the geometric mean fluorescence index).

### Construction of a *Streptococcus mitis pspC*^+^ Strain

For heterologous expression of *S. pneumoniae pspC* by *S. mitis*, *pspC* was inserted downstream of the lactate dehydrogenase (*ldh*) gene under the control of the native promoter. RNAseq data has shown *ldh* is highly expressed constitutively (unpublished data). An antibiotic resistance cassette for kanamycin (*km*^*r*^) was inserted into an intergenic spacer (IGS) site upstream of *pspC* within the *S. pneumoniae* TIGR4 strain genome. Small flanking fragments either side of the IGS in *S. pneumoniae* were amplified (primers: 885/886, 887/888) (Table 2), and ligated to *km*^*r*^ using separate bipartite ligations and then overlap extension PCR (primers 885 and 888) to generate a whole construct consisting of both 5′ and 3′ flanking regions and *km*^*r*^. This construct was then used to transform *S. pneumoniae* TIGR4. Small flanking fragments either side of the IGS downstream of *ldh* in *S. mitis* were amplified (primers: 875/876, 879/889) and then ligated to *pspC km^*r*^* amplified from the *S. pneumoniae* mutant. Two separate bipartite ligations were then again independently performed, and the two resulting ligation products linked by overlap extension PCR amplification (primers 875 and 889) to generate a construct consisting of both 5′ and 3′ flanking fragments, *pspC* and *km*^*r*^. This ligation product was then used to transform *S. mitis* SK142 (Supplementary Figure 1).

**TABLE 2:** Primers used during this study.

**Primer name**	**Sequence**
875	TGTTGCTGCTAACCCAGTTG
876	AATCTAGACCTGATGACTCAAGGAGTTATTTCT
879	AGGCCGGCCGTCTGTAGCAAGCAGTATTTGAC
885	TCCAAAAACAGGCTGGAAAC
886	AGGCGCGCCGCGCTATAAATCCGGCTAGA
887	AGGCCGGCCAATGGCGGCATTCAAGAG
888	TCAACAAGGTCCAGTCTGTCC
889	CAATCGACAAGAACGCTCAA

### Transformation

Transformation was carried out as previously described (Rukke et al., 2014) with minor modifications. Briefly, *S. mitis* pre cultures were diluted 1:12 in TSB and allowed to grow to an OD_600_ 0.03–0.04 prior to the addition of 5 mg DNA and competence stimulating peptide (CSP) (EIRQTHNIFFNFFKRR; GenScript) at a final concentration of 250 nM. The cultures were then incubated under aerobic conditions for 4 h at 37°C, 5% CO_2_ before selection on blood agar plates supplemented with the appropriate antibiotics.

### Whole Cell Lysate Preparation and Western Blotting

Bacterial strains were grown in THY medium until OD_580_ 0.6. Cells were then pelleted by centrifugation for 20 min at 3,000 rpm, washed twice in PBS, before the cell pellet was sonicated on ice using three 30 s bursts at 200–300 W. Cell lysates were then centrifuged to pellet all cellular debris, and protein concentrations measured in the supernatant using the BioRad DC^TM^Protein Assay. 6.25 μl of LDS Sample Buffer (4X) and 2.5 μl of Sample Reducing Agent (10X) was added to a known lysate concentration, making up to total volume of 25 μl using water, denatured at 70°C for 10 min prior to analyses using SDS-PAGE and Western blotting using pre-prepared 4–12% Bis-Tris Plus SDS-PAGE gels. Electrophoresis was carried out using 1X MES running buffer at a constant 200 V for 30 min, and the protein gels were transferred to PVDF membrane using a dry transfer iBlot system. Membranes were blocked in 5% milk in 1X TBS-Tween (TBS-T) for 2 h at room temperature, then incubated in primary antibody (1 h at room temperature or overnight at 4°C). Membranes were washed 3 times in TBS-T for 45 min and then incubated in secondary antibody for 1 h at room temperature. After washing again 3 times, membranes were developed using Luminata^TM^Crescendo HRP substrate (Merck Millipore) and imaged using the ImageQuant LAS 4000.

### Animal Infection Models

CD1 (female, 8–12 weeks) or C57BL/6 C3- (female, 14–15 weeks) mice were injected intraperitoneally with varying CFU suspended in 100 μl PBS. Blood and spleen were obtained from mice sacrificed at set time points (1, 4, 24 h), and serial dilutions of blood and spleen homogenates plated onto blood agar plates to enumerate bacterial CFU. In select experiments, Ly6G + neutrophils were depleted using 600 μg anti-ly6G monoclonal antibody (1A8, BioXCell) administered intraperitoneally in a 200 μl volume 24 h prior to bacterial challenge; we have previously shown this treatment reduces neutrophils recruited to lavage fluid by 94.8% 24 h post-infection (Wilson et al., 2015, 2017).

### Competitive Index Calculation Method Statement

The relative virulence of the strains is presented as a competitive index (CI), defined as the ratio of the test strain (single or double mutant strain) to the reference strain (wild-type or a single mutant strain) recovered from the mice divided by the ratio of the test strain to the reference strain in the inoculum. A CI of < 1.0 indicates that the test strain is reduced in virulence compared with the reference strain. The lower the CI the greater the reduction in virulence.

### Statistics

Statistical analysis was performed using GraphPad Prism 8.0. Data were presented as group means ± the standard error of the mean (SEM). Results expressed as means were compared using either one way ANOVAs with *post hoc* tests for multiple groups or Students *T*-test when comparing the mean of two groups only. *In vitro* data are representative of results obtained from at three or more repeated experiments.

### Ethics Statement

All *in vivo* experiments were carried out at the biological services unit at UCL according to United Kingdom national guidelines for animal use and care, and were approved by the UCL Biological Services Ethical Committee and the UK Home Office (Project Licence PPL70/6510).

## Results

### *S. mitis* Strains Have Increased Sensitivity to Complement Compared to *S. pneumoniae* Independent of Capsular Serotype

**FIGURE 1:**
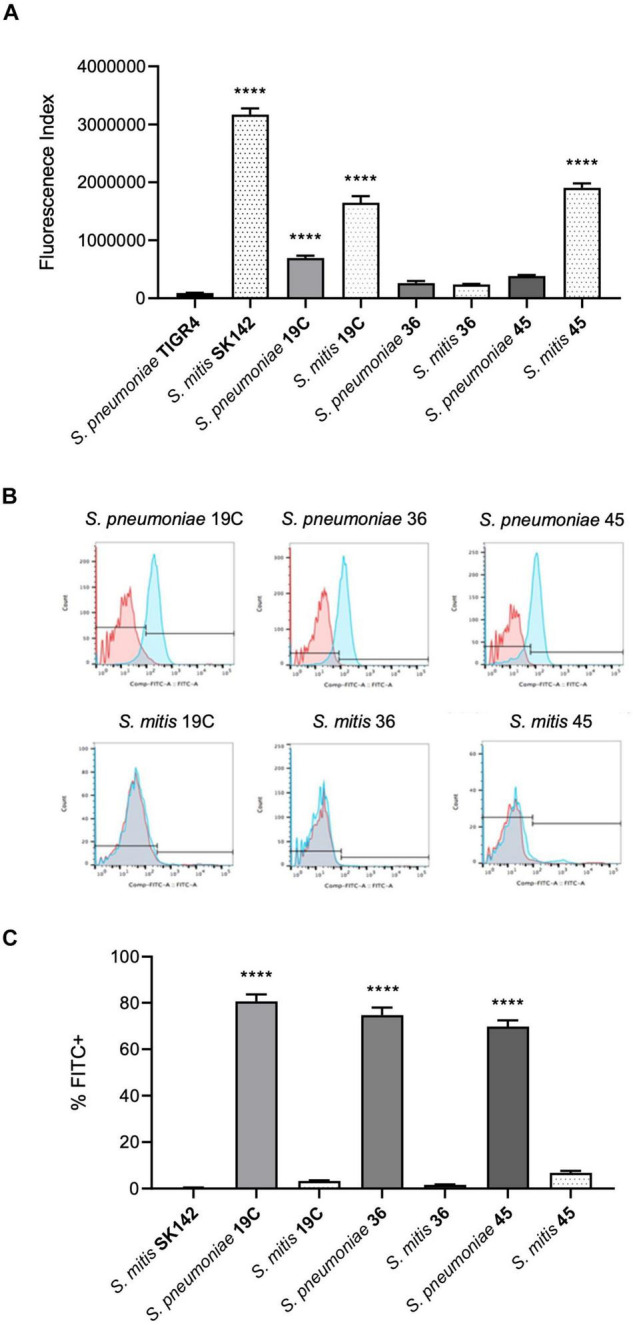
Comparison of C3b/iC3b deposition and ability to bind Factor H between S. pneumoniae and S. mitis. **(A)** Mean levels of C3b/iC3b deposition (shown as a fluorescence index) after incubation in 10% human serum and measured by flow cytometry. Data shown were analysed using a One-way ANOVA and a Dunn’s multiple comparisons test comparing the mean of each column with the mean of the control column. ****p < 0.0001. **(B)** Examples of flow cytometry histograms of Factor H binding to *S. pneumoniae* and *S. mitis*. **(C)** Corresponding analysed data showing the percentage of cells that were FITC positive of *S. pneumoniae* and *S. mitis* strains. Error bars represent SEM. Data were analysed using a One-way ANOVA and a Dunn’s multiple comparisons test comparing the mean of each column with the mean of the control column, *S. mitis* SK142. ****p < 0.0001.

To assess whether differences in sensitivity to complement independent of capsule expression may underpin the relatively low virulence of *S. mitis* strains we investigated C3b/iC3b deposition on four *S. pneumoniae* capsular serotypes (4, 19C, 36, and 45) and four *S. mitis* strains, three of which express *S. pneumoniae* capsular serotypes (19C, 36, and 45)(Figure 1A). The last *S. mitis* strain (SK142) expresses a capsule with an unknown chemical structure and serotype. C3b/iC3b deposition on the *S. mitis* and *S. pneumoniae* strains was measured using an established flow cytometry assay (Brown et al., 2002). With the exception of *S. mitis* serotype 36 (SK1126), there was significantly greater C3b/iC3b deposition on the *S. mitis* strains compared to the *S. pneumoniae* strains. Comparing between *S. mitis* strains, *S. mitis* SK142 was the most susceptible to complement deposition, with *S. mitis* serotype 36 being the least sensitive (Figure 1A). Amongst the *S. pneumoniae* strains, serotype 36 (1095/39) was again the most resistant to opsonisation with C3b/iC3b. These data indicate that *S. mitis* strains are more readily recognised by the complement system than *S. pneumoniae* strains even when they express a capsule, including capsules chemically identical to *S. pneumoniae* serotypes.

### Lack of Factor H Binding to the *Streptococcus mitis* Strains

One mechanism by which *S. pneumoniae* evades opsonisation with C3b is by PspC-mediated binding of FH (Dave et al., 2001). Homologues of *pspC* have not been found in the sequenced *S*. *mitis* genomes to date suggesting lack of PspC might be one reason why there are differences in complement sensitivity between *S. pneumoniae* and *S. mitis* strains expressing the same capsular serotype. We therefore assessed FH recruitment to the *S. mitis* strains after incubation in 10% human serum using flow cytometry. This demonstrated no evidence of significant factor H binding to any of the *S. mitis* strains whereas, as previously described, *S. pneumoniae* strains showed a high level of binding to factor H (Figures 1B,C).

### Expression of PspC Improves *Streptococcus mitis* Resistance to Complement-Mediated Immunity

**FIGURE 2:**
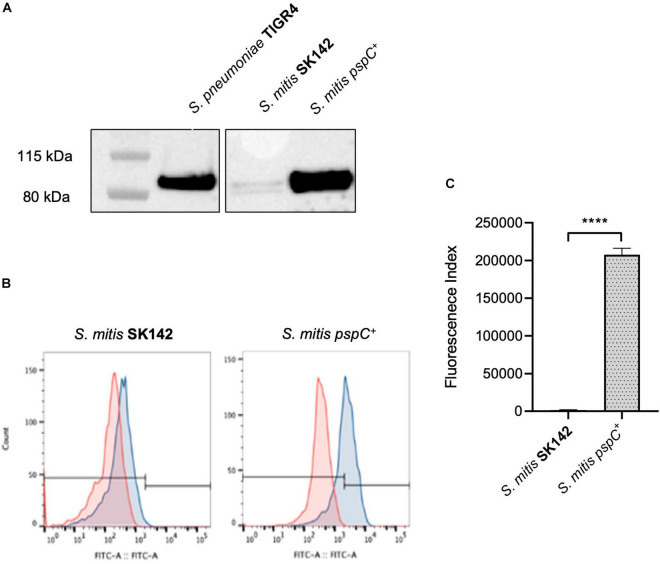
Construction and confirmation of *S. mitis* SK142 strain expressing *S. pneumoniae* TIGR4 *pspC*. **(A)** Western blot of whole bacterial lysates to demonstrate PspC protein expression, probed with a polyclonal anti-PspC antibody generated through immunisation of mice with PspC (kind gift of Sven Hammerschmidt). **(B)** Examples of Factor H binding flow cytometry histograms and **(C)** corresponding quantitative fluorescence data presented as mean fluorescence index for factor H binding. Error bars represent SEM and data were analysed using an unpaired *t*-test. ****p < 0.0001.

**FIGURE 3:**
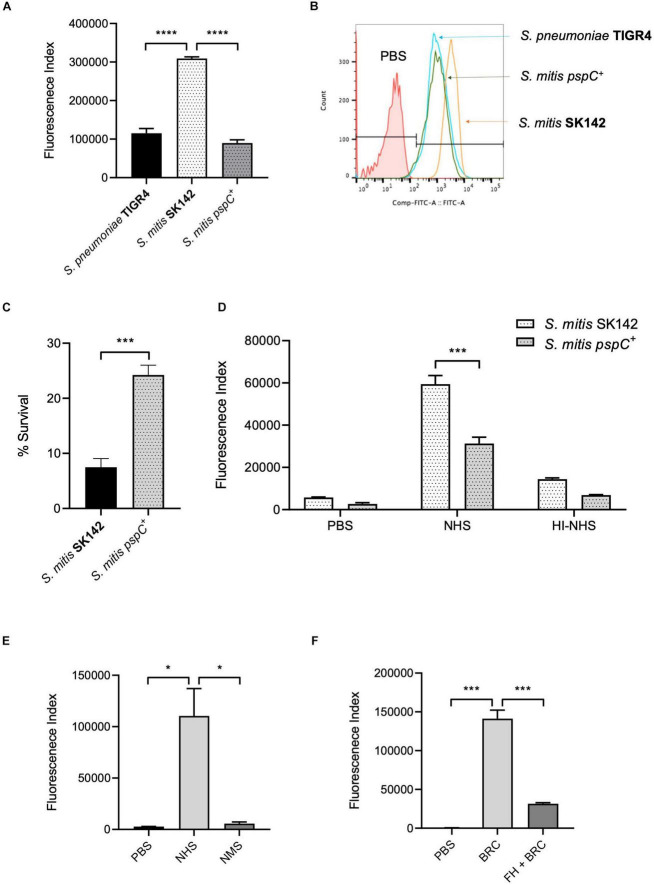
Effects of PspC on *S. mitis* survival. **(A)** Complement deposition on *S. pneumoniae* TIGR4, *S. mitis* wild-type and *S. mitis pspC^+^* measured by flow cytometry in normal human serum and **(B)** Representative flow cytometry histogram plot of complement C3/iC3b binding. Error bars represent SEM. Data were analysed using a One-way ANOVA and a Tukey’s multiple comparisons test. ****p < 0.0001. The line marked PBS represents the negative control, wild-type *S. pneumoniae* incubated in PBS alone. **(C)** Growth and survival of *S. mitis* compared to *S. mitis pspC^+^* in whole human blood. 5 × 10^6^ CFU/strain were added to 1 ml of fresh human blood and incubated at 37°C. Bacterial survival was determined following a 4-h incubation period and expressed as a percentage of the inoculum. Data expressed as mean, error bars represent SEM. Data shown was analysed using an unpaired two-tailed *t*-test. ***p < 0.001. **(D)** Effect of PspC expression in *S. mitis* on neutrophil opsonophagocytosis following incubation in 50% human serum and 50% heat-inactivated human serum. Error bars represent SEM. Data was analysed using a two-way ANOVA and a Tukey’s multiple comparisons test. ***p < 0.001. **(E)** Binding of factor H by *S. mitis pspC^+^* in human and mouse serum measured by flow cytometry. Error bars represent SEM. Data were analysed using a One-way ANOVA and a Tukey’s multiple comparisons test. *p < 0.05. **(F)** C3/iC3b deposition on *S. mitis pspC^+^* following incubation in 25% baby rabbit complement (BRC), with and without prior incubation in 100 μg/ml human factor H (FH + BRC). Data presented show mean and SEM, and were analysed using a One-way ANOVA and Tukey’s multiple comparisons test. ***p < 0.001.

The above data suggested increased sensitivity of *S. mitis* strains to complement independent of capsular serotype could be related to their inability to bind FH *via* PspC. To investigate this possibility, the *S. pneumoniae* TIGR4 strain *pspC* was expressed in *S. mitis* SK142. Immunoblots confirmed that the *S. mitis pspC^+^* expressed PspC protein (Figure 2A). Expression of *pspC* resulted in a reduction in growth levels over 8 h (Supplementary Figure 2) but demonstrated significant levels of FH binding to *S. mitis* (Figures 2B,C). The functional consequences of expression of *pspC* by *S. mitis* was assessed using *in vitro* assays of C3b/iC3b deposition, growth in blood, and neutrophil phagocytosis (Figure 3). Flow cytometry confirmed that the *S. mitis pspC^+^* strain was indeed more resistant to opsonisation with complement, showing similar levels of C3b/iC3b deposition as the *S. pneumoniae* TIGR4 strain (Figures 3A,B). The *S. mitis pspC^+^* strain showed increased survival in whole human blood compared to the parental wild type *S. mitis* strain, with an increase in CFU compared to the wild type of almost 150% after 4 h (Figure 3C). The *S. mitis pspC^+^* strain also demonstrated a reduced bacterial association with neutrophils when incubated in whole human serum (NHS), but not when incubated in PBS or in heat-inactivated human serum (Figure 3D), suggesting this effect was due to the reduced opsonisation of *S. mitis pspC^+^* with complement. These data demonstrate that functional PspC was successfully expressed by the *S. mitis pspC^+^* strain and, as predicted, increased the strain’s resistance to complement-mediated immunity.

### Role of Complement for Immunity to *Streptococcus mitis* Strains in Mouse Models of Infection

**FIGURE 4:**
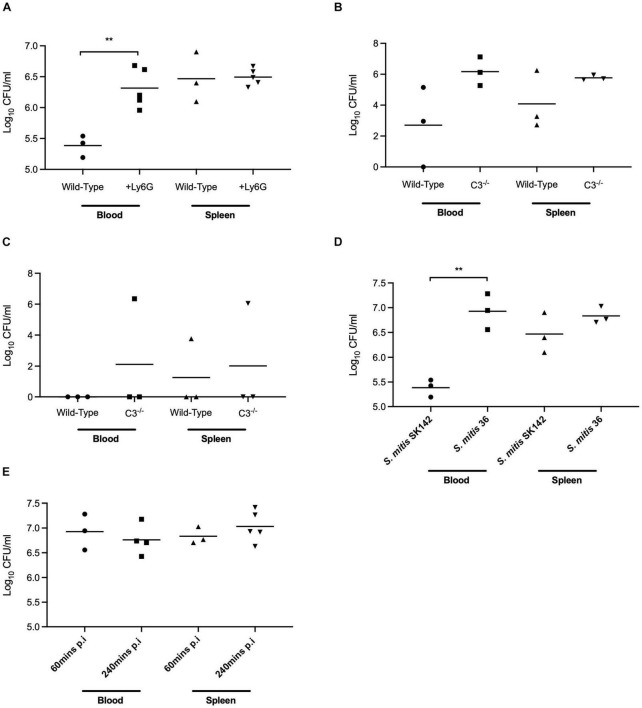
*In vivo* survival of *S. mitis* in mouse models of lung infection and bacteraemia. **(A)** *In vivo* survival of *S. mitis* SK142 following 1-h infection in neutrophil depleted CD1 mice. Neutrophils were depleted in mice using 600 μg of an anti-Ly6G + antibody 24 h prior to infection. Mice were inoculated intraperitoneally with 5 × 10^6^ CFU prior to a 1-h infection. Data were analysed using unpaired student *t*-tests. **p < 0.01. **(B)** *In vivo* survival of *S. mitis* SK142 in C57BL/6 C3^– /–^ mice following a 1-h intraperitoneal infection with 5 × 10^6^ CFU of the wild-type *S. mitis* SK142. Data were analysed using unpaired student *t*-tests. **(C)** *In vivo* survival of *S. mitis* SK142 in C57BL/6 C3^– /–^ mice following a 4-h infection. Complement deficient mice were infected by intraperitoneal injection with 5 × 10^6^ CFU of the wild-type *S. mitis* SK142 and culled at 1 h to obtain bacterial CFU in blood and spleen. Data were analysed using unpaired student *t*-tests. **(D)** *In vivo* survival of *S. mitis* strains following intraperitoneal infection of CD1 mice for 1-h with 5 × 10^6^ CFU. Blood and homogenised spleen were serially diluted and plated for bacterial enumeration. Data were analysed using a two-tailed unpaired *t*-test. **p < 0.01. **(E)** Comparison of *in vivo* survival of *S. mitis* ST36 following intraperitoneal infection of CD1 mice with 5 × 10^6^ CFU and sacrificed 4-h post-infection. The data for 1-h post infection are reproduced from Figure 6A and presented here for comparison. Data were analysed using unpaired student *t*-tests.

A mouse model of sepsis was used to investigate whether differences in complement sensitivity might contribute toward the relatively low virulence of *S. mitis*. In this model, the *S. mitis* SK142 strain is very rapidly cleared with a 99% reduction in bacterial CFU after 30 min (Rukke et al., 2014). As neutrophils mediate complement dependent immunity against streptococcal species we first assessed the importance of neutrophils for the rapid clearance of *S. mitis* in the mouse model of infection. Mice were depleted of neutrophils using an anti-Ly6G monoclonal antibody administered 24 h prior to infection. This causes an eightfold reduction in neutrophil numbers (Wilson et al., 2015). At 1 h post-intraperitoneal inoculation of 5 × 10^7^ CFU *S. mitis* SK142, blood CFU were approximately 10-fold higher in neutrophil depleted mice (6.3 log_10_ SD 0.32) compared to untreated mice (5.4 log_10_ SD 0.18, *P*-value 0.04 Student’s *t*-test), but no differences were seen in CFU recovered from the spleen (6.47 log10 SD 0.41, vs. 6.50 log10 SD 0.14, respectively; Figure 4A). These data suggest some contribution from neutrophils toward the rapid clearance of *S. mitis* from the blood during systemic infection of mice, and is compatible with a significant role for complement in this process. To investigate the potential role for complement further, infection experiments were repeated in mice deficient in complement factor C3. One hour after intraperitoneal inoculation with 5 × 10^6^ CFU of the *S. mitis* SK142, blood and spleen CFU were approaching 2 log_10_ higher in C3^–/–^ mice compared to wild-type mice, although the low n-number prevented these differences being statistically significant (Figure 4B). However, 4 h after inoculation, two out of three C3 deficient mice were still able to clear bacteria completely from the blood and spleen (Figure 4C).

To further investigate the role of complement during actual *S. mitis* infection, mice were inoculated intraperitoneally with a high dose (5 × 10^7^ CFU) of *S. mitis* SK142 and the relatively complement resistant *S. mitis* serotype 36 strain. Bacterial CFU were obtained from blood and spleen after 1 h to identify differences between strains. Mice inoculated with *S. mitis* serotype 36 showed outward signs of mild sickness (ruffled fur, hunched posture, and reduced mobility) that were not observed in mice infected with the SK142 strain, and also had approximately 100-fold more CFU in the blood after 1 h (around 10^7^ per ml) (Figure 4D). There was no significant difference seen between strains in the CFU recovered from the spleen. Repeat experiments showed persistence of serotype 36 *S. mitis* CFU 4 h after inoculation (Figure 4E), a timepoint when SK142 has been almost completely cleared from both blood and spleen (Figure 4C). Hence, the complement resistant serotype 36 *S. mitis* strain showed increased resistance to rapid bacterial clearance in mice compared to the more complement sensitive *S. mitis* SK142 strain, suggesting a role for complement in early clearance of *S. mitis* from the blood. However, by 24 h there was complete clearance of the serotype 36 *S. mitis* strain from both the blood and spleen (data not shown) demonstrating that even this relatively complement resistant *S. mitis* strain was unable to establish sustained infection.

To further assess the role of complement for controlling *S. mitis* CFU in the mouse model of sepsis we investigated whether the increased complement resistance of the SK142 *S. mitis pspC^+^* strain resulted in a reduction in the usual rapid clearance of the SK142 strain in the sepsis model. Previously it has been shown that *S. pneumoniae* PspC (Dave et al., 2001) does not bind mouse FH, and compatible with that observation no mouse FH binding was identified to the *S. mitis pspC^+^*strain incubated in mouse serum (Figure 3E). Hence to investigate whether human factor H could protect *S. mitis pspC^+^* from complement during mouse infection, bacteria were pre-incubated in purified human FH before inoculation into mice. *In vitro* experiments demonstrated that pre-incubation in human FH bound by *S. mitis pspC^+^* inhibited C3b/iC3b deposition when the bacteria were subsequently incubated in baby rabbit complement (Figure 3F). For the *in vivo* experiments, we used competitive infection of the *S. mitis pspC*^+^ strain with the *S. mitis* wild-type (complement sensitive) strain as this is highly sensitive at identifying differences in virulence between bacterial strains (Yuste et al., 2005). However, after pre-incubation in human FH the *S. mitis pspC*^+^ strain had no competitive advantage with a mean CI of 0.9 (SD 0.23) 1 h post intraperitoneal injection. These data indicate that increased resistance to complement does not overcome the rapid clearance of *S. mitis* in the mouse model.

### Poor Survival and Growth of *Streptococcus mitis* Strains in Biological Fluids

**FIGURE 5:**
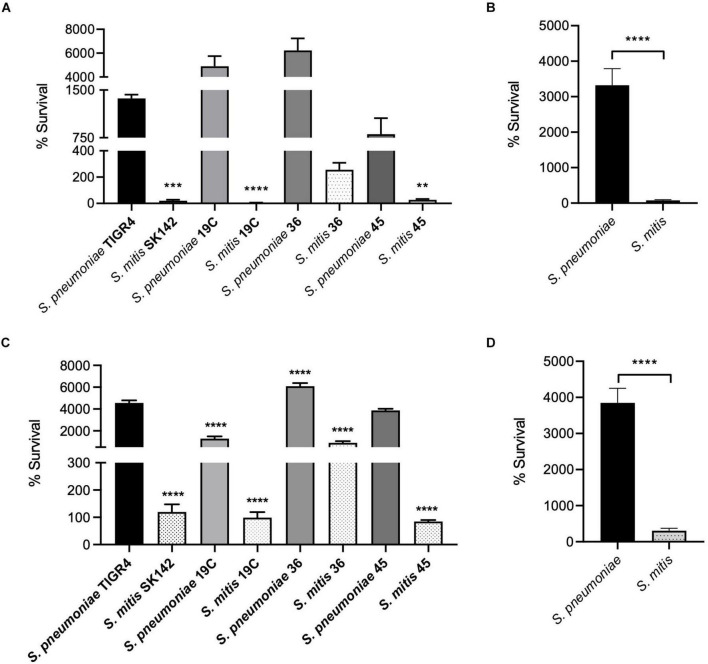
Growth and Survival of capsule-matched *S. pneumoniae* and *S. mitis* strains. **(A)** *In vitro* survival of *S. pneumoniae* and *S. mitis* with varying capsule serotypes in whole human blood. 5 × 10^6^ CFU/strain was added to 1 ml fresh human blood and incubated at 37°C. Survival in blood was determined following a 4-h incubation period. Bars represent percent change in the number of CFU/ml^– 1^ in relation to the inoculum and error bars shown represent SEM. Survival of less than 100 represents bacterial killing, whereas % survival greater than 100 represents bacterial growth. Data shown were analysed using a One-way ANOVA and a Dunn’s multiple comparisons test comparing the mean of each column with the mean of the *S. pneumoniae* TIGR4 control column. **p < 0.01, ***p < 0.001, ****p < 0.0001. **(B)** Average survival data for both bacterial strains in whole human blood. Error bars represent SEM. Data shown were analysed using a two-tailed *t*-test. ****p < 0.0001. **(C)** *In vitro* growth and survival in heat-inactivated (HI) human serum. 5 × 10^6^ CFU/strain was inoculated into 1 ml heat-inactivated human serum and incubated at 37°C. Survival was determined following a 4-h incubation period. Bars represent percent change in the number of CFU/ml^– 1^ in relation to the inoculum, error bars shown represent SEM. Survival of less than 100 represents bacterial killing, whereas % survival greater than 100 represents bacterial growth. Data shown were analysed using a One-way ANOVA and a Dunn’s multiple comparisons test comparing the mean of each column with the mean of the *S. pneumoniae* TIGR4 control column. ****p < 0.0001. **(D)** Average survival data for both bacterial strains in heat-inactivated human serum. Error bars represent SEM. Data shown were analysed using a two-tailed *t*-test. ****p < 0.0001.

As replication under conditions encountered during infection is essential for bacterial virulence independent of the ability to evade innate immunity, we compared the growth in blood and serum of the *S. pneumoniae* and *S. mitis* strains. Three of the four *S. mitis* strains studied had poor survival in blood, with < 50% of the inoculum CFU recovered after 4 h incubation (Figure 5A). The exception was *S. mitis* serotype 36 which increased CFU by approximately 100% compared to the inoculum. However, this increase in CFU was markedly lower than that seen for the *S. pneumoniae* strains, which all demonstrated a > 10-fold increase in CFU. The serotype 19C and serotype 36 had particularly large increases in CFU after 4 h incubation in blood with a > 40-fold increase. These data indicate that the *S. mitis* strains have an impaired ability to replicate in blood compared to *S. pneumoniae* (Figure 5B), and that this effect is independent of capsular serotype as two of the *S. mitis* strains expressing *S. pneumoniae* capsular serotypes were still unable to survive in blood. To determine whether the low survival rates of *S. mitis* in blood was due to the action of persisting cellular and soluble immune effectors within the blood (e.g., complement and neutrophils), or due to poor growth of *S. mitis* under physiological conditions, growth of all strains in heat-inactivated (HI, to deplete complement activity) human serum was assessed (Figure 5C). Survival of all four *S. mitis* strains significantly increased in HI-sera compared to blood, with all strains demonstrating at least maintenance of inoculum CFU. Again, higher levels of *S. mitis* ST36 were recovered in comparison to the other three strains. However, the *S. pneumoniae* strains still grew considerably better in serum than the *S. mitis* strains, with only a marginally smaller difference in percentage survival between the pooled data for all four *S. pneumoniae* strains compared to the *S. mitis* strains in serum compared to blood (Figure 5D). These results suggest that although the presence of cells and active complement in the human blood could be exacerbating the differences in survival between *S. mitis* and *S. pneumoniae*, differences in growth rates in physiological fluid had a more dominant effect on differences in replication rates between *S. mitis* and *S. pneumoniae.*

## Discussion

Although *S. pneumoniae* and *S. mitis* are genetically closely related and share a substantial number of virulence factors, there is a significant disparity in pathogenicity between these two species. Evasion of complement-mediated immunity is important for *S. pneumoniae* virulence, and differences in susceptibility to complement correlates with the invasive potential of different *S. pneumoniae* strains (Hyams et al., 2013). Hence, our finding that *S. mitis* strains are more susceptible to complement than *S. pneumoniae* strains could be one reason underpinning the relatively low virulence of *S. mitis*. These data provide a genetic basis why *S. mitis* is more susceptible to complement than *S. pneumoniae*, although the data on complement resistance needs extending to a larger set of strains representative of the overall genetic diversity of *S. mitis* and *S. pneumoniae* to confirm that there is a significant difference in complement resistance between these species.

Mouse infection experiments were used to assess whether complement was an important component as to why *S. mitis* fails to establish infection in mice after intraperitoneal inoculation (Rukke et al., 2014). Neutrophil-mediated clearance of streptococci is highly complement dependent (Marshall et al., 2019); hence the increase in recovered CFU from mice after neutrophil depletion, combined with the data indicating the complement resistant *S. mitis* strain ST36 survives better *in vivo* compared to SK142 indicate there is a significant role for complement in the rapid clearance of *S. mitis* from the blood and spleen in the mouse model. However, both the serotype 36 and SK142 *S. mitis* strains were completely cleared from blood and spleen by 24 h, demonstrating that other factors to complement have dominant roles in preventing *S. mitis* establishing infection in mice. Although the data are weakened by the limited availability of C3^–/–^ mice and low *n* number, experiments using C3^–/–^ mice also suggested there was an early effect of complement but this was not the dominant factor underpinning the rapid clearance of *S. mitis* from the mice.

*S. pneumoniae* has only limited factors that are known to directly cause host damage, and instead, *S. pneumoniae* virulence is largely dependent on highly effective mechanisms of immune evasion including inhibition of complement pathways (Hyams et al., 2013; Camberlein et al., 2015). Although the capsule is the major *S. pneumoniae* virulence factor and strongly affects susceptibility to complement (Hyams et al., 2010b, 2013), the differences in complement sensitivity between *S. mitis* and *S. pneumoniae* were present even when the strains expressed the same capsular serotype. Hence, the higher susceptibility to complement of *S. mitis* was not related to lack of a capsule. Instead, this phenotype could reflect the relative thickness of the capsule layer compared to *S. pneumoniae* (Marshall et al., 2019), and *S. pneumoniae* is known to be able to regulate capsule expression although the mechanisms are not well understood (Kilian and Tettelin, 2019). In addition, *S. mitis* could lack one or more of the multiple protein factors involved in complement evasion by *S. pneumoniae*, including pneumolysin and multiple surface proteins (Yuste et al., 2005; Hyams et al., 2013; Brown et al., 2015). By comparing genomic data, Kilian and Tettelin (2019) identified which of 224 known *S. pneumoniae* virulence genes were absent in closely related commensal species and therefore may play a role in their different pathogenic potentials. Of these, three genes known to play a role in *S. pneumoniae* complement evasion were either not present or uncommon in *S. mitis*; *pspC* (binds FH to inhibit alternative pathway activation and present in 0% of *S. mitis* genomes, investigated here), *pspA* (impairs alternative pathway recognition of *S. pneumoniae*, present in 0% of *S. mitis* genomes), and *ply* (encodes the toxin pneumolysin which impairs classical pathway recognition of *S. pneumoniae*, present in 15% of *S. mitis* genomes). Mitilysin has approximately 15 amino acid substitutions compared to pneumolysin (Jefferies et al., 2007), and we are not aware of any data on its role in preventing complement activation by *S. mitis.*

Unlike the *S. pneumoniae* strains, the wild-type *S. mitis* strains used in this study did not bind the negative regulator of complement activity FH. To investigate whether this contributed to lack of *S. mitis* complement resistance we successfully expressed PspC in the *S. mitis* strain SK142. Expression of PspC in *S. mitis* retained its functional role of binding FH and resulted in a significant reduction in the amount of complement bound to *S. mitis*, prevented complement-dependent neutrophil phagocytosis, and increased survival when cultured in blood. These phenotypes are all consistent with an increased ability of the *S. mitis pspC^+^* strain to evade complement-mediated neutrophil killing. Although a detailed assessment of the effects of PspC expression on *S. mitis* virulence is complicated by the species-specificity of the PspC/factor H interaction, when tested by competitive infection experiments after pre-incubation in human FH there was no difference in virulence in mice between the wild type and *pspC ^+^ S. mitis* strains. This result again suggests complement was not the dominant reason for the lack of virulence of *S. mitis* in mice. PspC is also involved in transmigration of epithelial and endothelial barriers, and future work will address whether expression of *pspC* in *S. mitis* alters interactions with host cells (Orihuela et al., 2009; Hyams et al., 2010b, 2013; Iovino et al., 2017).

The above results indicated other factors to complement sensitivity are involved in why *S. mitis* is unable to establish sustained infection in mice. Bacterial replication and survival in blood is an essential step for a pathogen in establishing disease. Hence, we compared *in vitro* growth in human blood and serum for the *S. mitis* and *S. pneumoniae* strains and demonstrated marked differences between the species. The *S. mitis* strains exhibited poor growth and survival in human blood, whereas the *S. pneumoniae* strains exhibited substantial growth. In heat-treated serum *S. mitis* survival improved but in general this remained markedly lower than that of the *S. pneumoniae* strains. Hence the poor growth of *S. mitis* in blood and sera seems to reflect two factors; a greater susceptibility to immune mechanisms and a growth defect in the physiological conditions found in blood. Poor growth in blood is likely to be a strong contributory factor as to why *S. mitis* is so rarely found as a cause of bacteraemia.

To conclude, comparing complement sensitivity of four *S. mitis* and four *S. pneumoniae* encapsulated strains has demonstrated *S. mitis* is unable to bind FH and is much less resistant to complement than *S. pneumoniae*. Expression of the *S. pneumoniae pspC* by *S. mitis* resulted in FH binding to *S. mitis* and reduced this strains sensitivity to complement. However, the inability of *S. mitis* to establish infection in mice was not strongly dependent on complement sensitivity, and instead was more likely related to the inability of *S. mitis* strains to replicate rapidly in serum. Why *S. pneumoniae* grows rapidly in serum whereas *S. mitis* does not is likely to have a multifactorial basis that will require further investigation.

## Data Availability Statement

The raw data supporting the conclusions of this article will be made available by the authors, without undue reservation.

## Ethics Statement

The animal study was reviewed and approved by the all blood and sera collection was approved by the UCL Research Ethics Committee (Ref:3076/001) *in vivo* experiments were approved by the UCL Biological Services Ethical Committee and the United Kingdom Home Office under project licence (PPL 70/7361).

## Author Contributions

HM, FP, and JB were involved in the proof-of concept and experimental design of this study. HM performed all experiments with support from RJ. MK provided essential materials. HM, RJ, FP, MK, and JB wrote, edited and proofread the manuscript. All authors contributed to the article and approved the submitted version.

## Conflict of Interest

The authors declare that the research was conducted in the absence of any commercial or financial relationships that could be construed as a potential conflict of interest.

## Publisher’s Note

All claims expressed in this article are solely those of the authors and do not necessarily represent those of their affiliated organizations, or those of the publisher, the editors and the reviewers. Any product that may be evaluated in this article, or claim that may be made by its manufacturer, is not guaranteed or endorsed by the publisher.
