# Supplementary figures and images for: Effects of Expression of Streptococcus pneumoniae PspC on the Ability of Streptococcus mitis to Evade Complement-Mediated Immunity
Source: Front Microbiol. 2021 Nov 22;12:773877. doi: 10.3389/fmicb.2021.773877 (PMC8646030; doi:10.3389/fmicb.2021.773877)

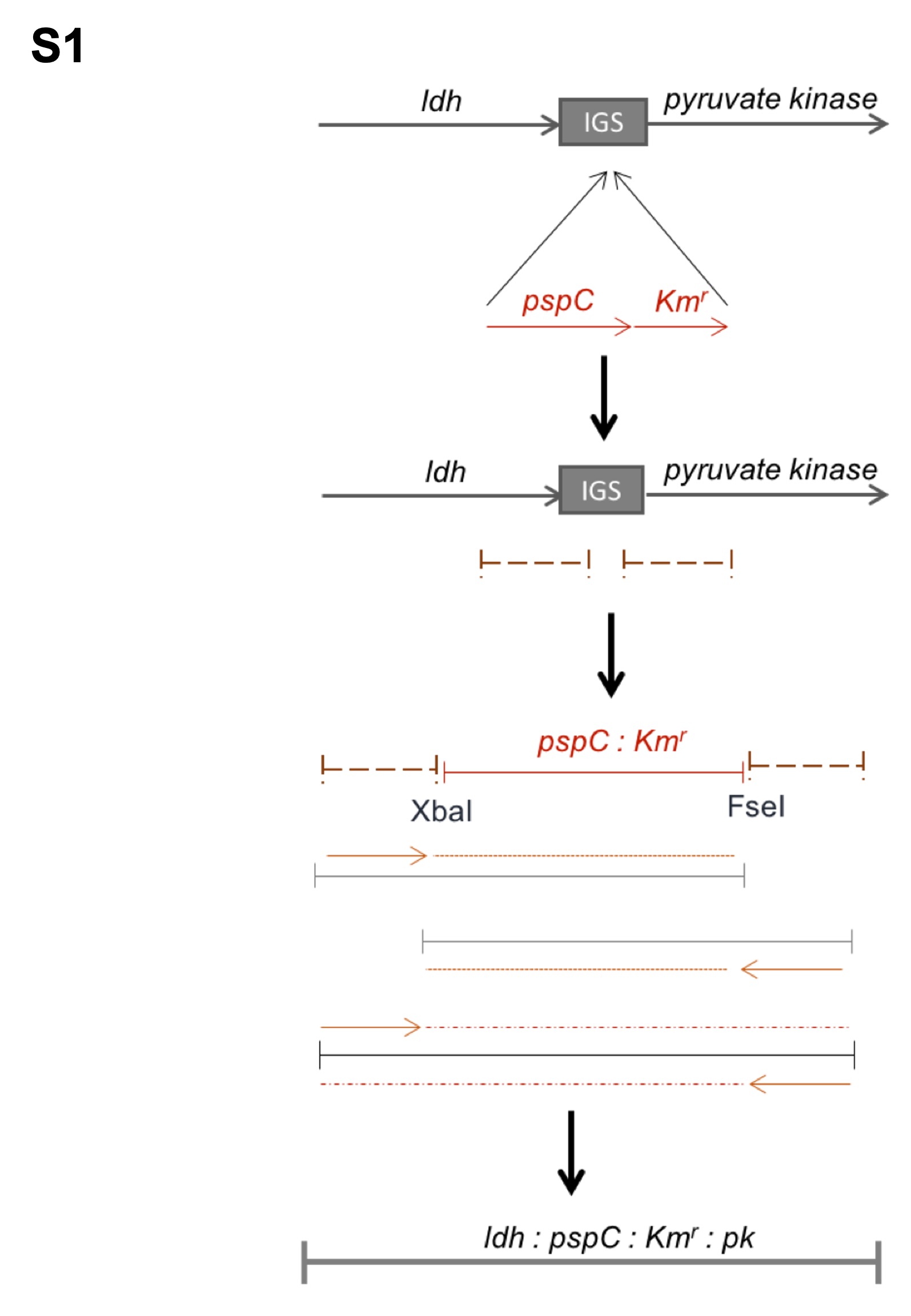

Supplement: Supplementary file 1 [file Image_1.JPEG]

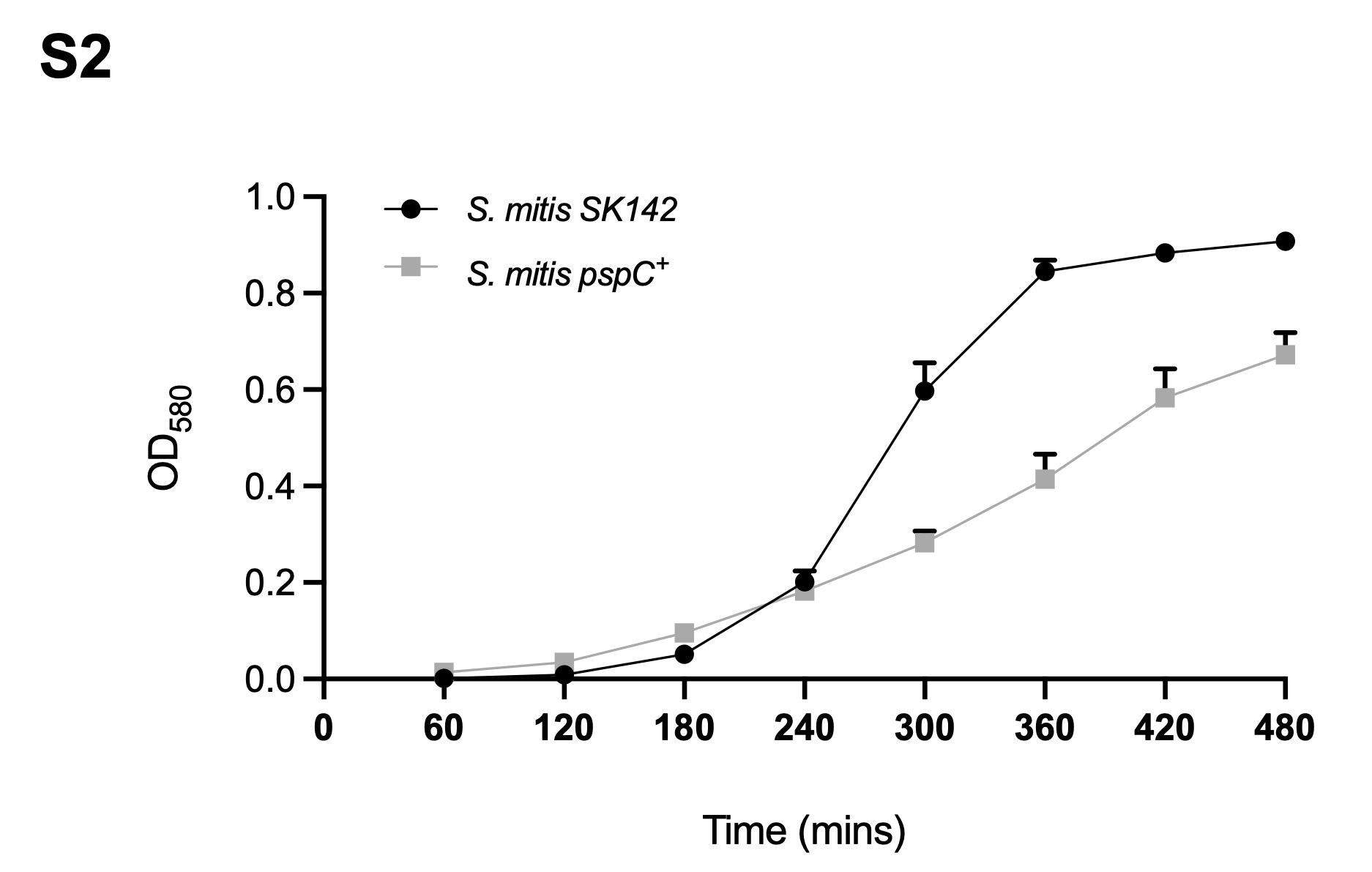

Supplement: Supplementary file 2 [file Image_2.JPEG]
